# The Impact of Coping Flexibility on the Risk of Depressive Symptoms

**DOI:** 10.1371/journal.pone.0128307

**Published:** 2015-05-26

**Authors:** Tsukasa Kato

**Affiliations:** Department of Social Psychology, Toyo University, Tokyo, Japan; Carleton University, CANADA

## Abstract

**Objective:**

According to the dual-process theory, coping flexibility is defined as the ability to produce and implement a new coping strategy in place of an ineffective coping strategy. Specifically, coping flexibility includes two processes: evaluation coping and adaptive coping. Evaluation coping refers to sensitivity to feedback about the efficacy of a coping strategy, and adaptive coping involves the willingness to implement alternative coping strategies. The coping flexibility hypothesis (CFH) postulates that more flexible coping will be associated with more adaptive outcomes; importantly, there are numerous theories and studies that support the CFH. The main purpose of this study was to test the CFH based on dual-process theory.

**Methods:**

A total of 1,770 Japanese college students participated and, completed a set of questionnaires that measured coping flexibility (evaluation coping and adaptive coping) and depressive symptoms. Depressive symptoms were measured via the Center for Epidemiologic Studies Depression Scale.

**Results:**

The proportions of women and men who reported depressive symptoms were 58.69% (95% CIs [55.74, 61.66]) and 54.17% (95% CIs [50.37, 57.95]), respectively when a cut-off score of 16 on the CES-D was used. A multivariable logistic regression analysis revealed that evaluation coping (OR = 0.86, 95% CIs [0.83, 0.0.89]) and adaptive coping (OR = 0.91, 95% CIs [0.88, 0.93]) were significantly associated with lower levels of depressive symptoms.

**Conclusion:**

The results of the present study indicated that the CFH based on dual-process theory was supported in a Japanese sample.

## Introduction

Depression is a leading cause of disability worldwide. In interviews conducted by the World Health Organization (WHO) [1] with a community sample comprised of 60,463 adults, the prevalence of mood disorders ranged from 0.8% in Nigeria to 9.6% in the United States. Furthermore, the WHO [2] reported that unipolar depressive disorders were the leading cause of burden among all disease. Specifically, unipolar depressive disorders accounted for 8.6% of total disability-adjusted life years (DALYs), and was the leading cause of years of life lived with disability (YLDs), or 16.4% of YLDs.

The WHO [2] states that certain of types of mental and behavioral disorders, such as depression and anxiety, can occur as a result of failing to adaptively cope with stress. According to the transactional theory proposed by Lazarus and colleagues [3,4], coping behavior, which is defined as “constantly changing cognitive and behavioral efforts to manage specific external and/or internal demands that are appraised as taxing or exceeding the resources of the person” (p. 141), affects psychological adjustment and maladjustment, including depressive symptoms. This hypothesis has been supported by numerous studies [3]. However, the conventional coping research is limited by solely focusing on the way a specific coping strategy affects psychological dysfunction and well-being [5], and failing to consider the diversity and fluidity of coping behavior [5,6]. However, recent research and theories on flexibility suggest that no single behavior or strategy is always maximally adaptive [7,8]. Therefore, coping flexibility has been the subject of several research studies in recent decades [7,9].

Coping flexibility refers to one’s ability to effectively modify one’s coping strategies according to the demands of different stressful situations [6]. For example, Kato [6] defined coping flexibility as “the ability to discontinue an ineffective coping strategy and produce and implement an alternative coping strategy” (p. 263). Two coping processes were proposed based on this definition: evaluation coping and adaptive coping. According to the dual-process theory of coping flexibility [6], evaluation coping occurs when individuals abandon a coping strategy that produces undesirable outcomes; this process subsumes various strategies, including the comprehension of one’s environment, monitoring and evaluating coping outcomes, and abandoning an ineffective coping strategy when the outcomes prove unfavorable [6]. Adaptive coping refers to the consideration of alternate strategies and to their subsequent implementation after an ineffective strategy has been abandoned [6].

Kato’s [6] definition of coping flexibility involves a core process of flexibly coping with stressors. Cheng et al. [9] synthesized previous work on concepts of coping flexibility, and stated that a flexible coping process occurs in three main stages: planning, execution, and feedback. The planning stage is a process that selects the most appropriated strategy for handling a stressful situation. The execution stage is composed of *evaluation* and *adaptation* processes. Finally, in the feedback state, the efficacy of a chosen strategy is monitored. In Cheng et al.’s model of coping flexibility, three meta-coping skills—evaluation, adaptation, and monitoring—play a major role in the execution and feedback stages. Cheng’s model represents an integration of multiple concepts and theories of coping flexibility. Specifically, Kato [6] proposed that the evaluation and adaptation processes are part of the execution stage; moreover, the feedback process [7–9] was included in the definition of coping flexibility outlined in dual process theory. In addition, the concept of meta-coping was introduced into Kato’s definition [6] of coping flexibility. Therefore, the concept of coping flexibility in dual-process theory can be viewed as an execution process that includes a feedback function and a core process of flexibly coping with stressors.

There are numerous theories and studies that support the hypothesis that more flexible coping results in more adaptive outcomes (for reviews, see [6,9]); this is generally referred to as the coping flexibility hypothesis (CFH) [5,6]. In support of the CFH, Cheng et al. [9] conducted a meta-analysis examining the relation between coping flexibility (including Kato’s [6] definition) and psychological adjustment. The results from this study showed that the mean effect size (*r*) was .32 (95% confidence interval (CI) [.26, .37], *k* = 108, *N* = 28,145). For example, evaluation coping and adaptive coping were more strongly associated with lower depressive symptoms longitudinally [6]; this was beyond popular coping strategies and coping flexibility measured by other approaches. Although few studies [5,6,10] have tested the CFH based on the dual-process theory of coping flexibility, the CFH may be applicable to Kato’s [6] dual-process theory. Therefore, in the present study, we tested the CFH based on the dual-process theory of coping flexibility with a relatively large sample.

## Materials and Methods

### Participants and procedure

Participants included 1,770 Japanese college students (1,087 women and 683 men; *M* = 19.33, SD = 1.18, range = 18 to 26 for age). Seven participants were older than 26 years of age and were eliminated from the sample given that the strength of the relationship between coping flexibility and psychological adjustment differs with age (for a meta-analytic review, see [9]). After providing informed consent, participants completed questionnaires that assessed coping flexibility and depressive symptoms.

All procedures followed were in accordance with the ethical standards of the responsible committee on human experimentation (institutional and national) and with the Helsinki Declaration of 1975, as revised in 2000. The project was approved by the Institutional Ethnics Committee of the Department of Social Psychology at Toyo University in Japan. All participants were provided their verbal and written informed consent to participate in the current study.

### Measures

The measures that were originally in English were translated into Japanese; the reliability and validity of the Japanese versions were estimated by Kato [6].

#### Coping flexibility

The Coping Flexibility Scale (CFS) [1] is based on the dual-process theory of coping flexibility; this measure was used to measure coping flexibility. The CFS consists of two subscales; evaluation coping (e.g., If I feel that I have failed to cope with stress, I change the way in which I deal with stress) and adaptive coping (e.g., When a stressful situation has not improved, I try to think of other ways to cope with it); each subscale has five items. Confirmatory factor analyses (CFAs) demonstrated that there was a good fit to the two-factor model for the CFS; the fit to the other models was poor [6]. In addition, each coping score was associated with theoretically related constructs and predicted higher scores on an insight problem-task that requires flexible thinking. Importantly, each subscale score for the CFS were not associated with scores for social desirability and self-confidence during a task [6]. Moreover, the CFH has been supported by some studies using the CFS in samples of chronic pain patients [5], employees [6], and college students [6,10]. Across 11 samples in Japan, Cronbach’s alphas for evaluation coping ranged from .71 to .91, and ranged from .83 to .90 for adaptive coping [6]. Participants rated the extent to which each item applied to them on a 4-point scale ranging from 0 (not applicable) to 3 (very applicable).

#### Risk of depressive symptoms

The Center for Epidemiologic Studies’ Depression Scale (CES-D) [11] was used to estimate the risk of depressive symptoms in this study. This scale comprises 20 items: 16 negatively oriented items and 4 positively oriented items. The development of the CES-D was originally based on an American population [11]; however, the validity and reliability of CES-D scores have also been established with a Japanese population [12]. Participants rated each item according to their experiences within the past week on a 4-point Likert scale ranging from 0 (rarely or none of the time, less than 1 day) to 3 (most or all of the time, 5–7 days).

In the current study, a CES-D score of 16, the traditional cut-off point for this scale, was used to identify a sample of depressed college students. This cut-off point was also recommended for Japanese samples [12]. However, the CES-D scores reported with Japanese samples are often higher than those in other countries. Specifically, the North West Adelaide Health Study [13] reported that the prevalence of depressive symptoms for 3,057 Australian university students was 12.4% when using the same cut-off point on the CES-D. However, a study of university students in Japan [14] indicated that the prevalence of depressive symptoms was 52.2% (95% CIs [46.5, 57.8]), and the mean CES-D score was 17.22 (SE = 0.53). Therefore, in the current study, two cut-off scores were used: 27 points and 16 points. The score of 27 was selected based on previous studies [15,16] that used a CES-D score of 27 as an indicator of moderate to severe depression.

## Results

Means, standard deviations, and Cronbach’s alphas for all variables are presented in Table 1. Mean scores for the CES-D for women and men were 20.42 (SD = 12.78, 95% CIs [19.66, 21.18]) and 18.73 (SD = 12.43, 95% CIs [17.80, 19.66]), respectively. The proportions of women and men with depressive symptoms using a CES-D score of 16 as the cut-off point were 58.69% (95% CIs [55.74, 61.66]) and 54.17% (95% CIs [50.37, 57.95]), respectively; thus, the prevalence of depressive symptoms was higher in women than in men (*B* = 0.18, SE = 0.10, Wald = 3.49, OR = 1.20, *p* = 0.064, 95% CIs [0.991, 1.458]). An analysis using a score of 27 as the cut-off point revealed that women (30.73%, 95% CIs [28.15, 33.45]) reported a significantly higher risk of depressive symptoms than men (25.04%, 95% CIs [21.87, 28.42]) did, *B* = 0.28, SE = 0.11, Wald = 6.64, OR = 1.33, *p* = 0.010, 95% CIs [1.07, 1.65].

**Table 1:** Means, Standard Deviations, and Alphas for Measures of Coping Flexibility and Depressive Symptoms.

Value	Mean	SD	Range	Alpha
Evaluation coping	9.51	3.13	0–15	0.69
Adaptive coping	7.01	3.42	0–15	0.88
Depressive symptoms	19.77	12.67	0–60	0.92

A multivariable logistic regression analysis was conducted to compute the adjusted odds ratios (ORs) associated with depressive symptoms, and the prevalence of depressive symptoms was computed with 95% CIs. The multivariable logistic regression analysis with a CES-D score of 16 used as the cut-off point revealed that evaluation coping (OR = 0.86, 95% CIs [0.83, 0.89], *p*< .001) and adaptive coping (OR = 0.91, 95% CIs [0.88, 0.93], *p*< .001) were significantly associated with a low risk of depressive symptoms, after controlling for gender (Table 2). The regression analysis with a score of 27 showed that evaluation coping (OR = 0.83, 95% CIs [0.80, 0.86], *p*< .001) and adaptive coping (OR = 0.96, 95% CIs [0.93, 0.99], *p* = .0023) were significantly associated with a low risk of depressive symptoms, after adjusting for gender (Table 2).

**Table 2:** Risk Factors of Depressive Symptoms.

						95% CI	
Risk Factor	B	SE	Wald	OR	*p* value	LL	UL
A 16 CES-D scores as the cut-off point							
Gender							
Men				1.00			
Women	0.12	0.10	1.42	1.13	0.233	0.92	1.39
Coping flexibility							
Evaluation coping	- 0.15	0.02	75.13	0.86	< 0.001	0.83	0.89
Adaptive coping	- 0.10	0.02	40.15	0.91	< 0.001	0.88	0.93
A 27 CES-D scores as the cut-off point							
Gender							
Men				1.00			
Women	0.27	0.11	5.36	1.30	0.021	1.04	1.63
Coping flexibility							
Evaluation coping	- 0.19	0.02	102.41	0.83	< 0.001	0.80	0.86
Adaptive coping	- 0.04	0.02	5.17	0.96	0.023	0.93	0.99

CES-D is the Center for Epidemiologic Studies’ Depression Scale; OR is odds ratio; CI is confidence interval for OR; LL is lower limit; UL is upper limit.

## Discussion

Evaluation coping and adaptive coping were significantly associated with a lower risk of depressive symptoms when scores of both 16 and 27 were used as the cut-off points on the CES-D, after adjusting for gender. The results indicated that the CFH based on dual-process theory was supported in this sample. Importantly, the sample size in the current study was large compared to previous studies on coping flexibility. In Cheng et al.’s [9] meta-analysis, only four of the 122 studies included in the meta-analysis had a sample size larger than 500 participants, and the study with the largest sample had 890 participants.

In the current study, women reported a higher risk of depressive symptoms than men when both cut-off points were used; however, the gender difference using a score of 16 was a non-significant trend. These results are consistent with the literature that indicates that women experience depression more often than men [17,18]. As expected, the prevalence of depressive symptoms in our sample was relatively higher than the rates reported in other countries; however, the prevalence of depressive symptoms in current sample was similar to that reported in other samples of Japanese college students using the same cut-off score (e.g., [14]).

There are known racial/ethnicity-specific response patterns on the CES-D [14]. Specifically, several studies [14,19] have suggested that some Asian and racial/ethnic groups, including Japanese individuals, have a tendency to inhibit the expression of positive affect on the CES-D, whereas Anglo-Americans have a tendency to overly express positively oriented items on the CES-D. Therefore, it is useful to also provide information about the negatively oriented items on the CES-D from the current study. This is significant given that the CES-D has been widely used in many countries and with many racial/ethnic groups [20]. Indeed, the article where the original version of the CES-D [12] was published was listed as 51^st^ (17,055 citations) out of 100 in a list of the most-cited papers of all time by *Nature* [21] in 2014. The means, standard deviations, and Cronbach’s alphas for the 16 negatively oriented items on the CES-D are shown in S1 Table. In addition, the results of the multivariable logistic regression analysis using a cut-off point of 13 for the 16 negatively oriented items [14] are also given in S2 Table. Importantly, the results were similar to the findings using the original version of the CES-D presented herein.

The findings from the current study that support the CFH could contribute to the development of stress management strategies that aid in attenuating depressive symptoms. This seems possible given that global prevention-oriented stress management programs are designed to aid in the acquisition of a repertoire of coping strategies, teach when and where strategies will be effective, and facilitate the selection of an appropriate strategy for a particular situation [22]. This type of stress management may be particularly helpful for individuals with chronic diseases or pain. Specifically, stress management may help them to attenuate depressive symptoms by providing information that will allow individuals to acquire skills to employ flexible coping. Indeed, many individuals with chronic diseases or chronic pain suffer from depressive symptoms [23,24]. Therefore, it is possible that these individuals engage in inflexible coping and require training to cope with stress more effectively. Indeed, the transactional theory states that the inability to successfully cope with stressors or recognize that a coping strategy is ineffective contributes to long-term dysfunction among those with chronic stress [3]. Therefore, it may be useful for those with chronic pain to acquire flexible coping rather than specific coping strategies targeting pain-related distress.

Coping flexibility has received considerable attention in recent decades [7,9], and researchers have measured coping flexibility based on multiple conceptualizations [6,9]. Nonetheless, researchers [6,9] have recently begun to integrate multiple approaches to the study of coping flexibility. However, a discussion of the nature of coping flexibility has not been sufficiently addressed in order to understand the relation between coping flexibility and adaptive outcomes. In the current study, we hypothesized that flexibility in coping would reduce depressive symptoms during stressful situations. However, individuals who possess more flexible coping in certain situations but have less flexible coping in other circumstances may produce more adaptive outcomes than those who consistently display flexible coping regardless of the situation. In other words, in a specific situation, engagement in a specific coping strategy may contribute to adaptive outcomes. In addition, research should be conducted on other types of flexibility, including cognitive flexibility, regulatory flexibility, and psychological flexibility, in order to examine if these types of flexibility promote more adaptive outcomes [6,7]; this type of research would contribute to an enhanced understanding of the relation between coping flexibility and adaptive outcomes. Importantly, Bonanno and Burton [7] proposed a framework that integrated coping flexibility with other types of flexibility; this framework may be useful to better understanding coping flexibility.

### Limitations

Despite the strengths of the current study, several limitations should be described, and some caution should be used in the interpretation of the findings. First, the findings cannot be generalized to other populations beyond Japanese college students. Indeed, Cheng et al.’s [9] meta-analysis showed that the relationship between coping flexibility and psychological dysfunction varies by culture. More specifically, it was reported that the link between coping flexibility and psychological adjustment was stronger for individuals from countries that are lower in individualism (e.g., Japan) than countries that are higher in individualism (e.g., United States). Second, a cross-sectional design using self-report measures was employed; therefore, a causal relationship between coping flexibility and a risk of depressive symptoms cannot be inferred. Although we hypothesized that inflexible coping would be one of the risk factors for depressive symptoms, it may also be that individuals with depression may employ more inflexible coping.

Although there were several limitations of the present study, the data from Japanese college students provide evidence that evaluation coping and adaptive coping were associated with a low risk of depressive symptoms. That is, the CFH based on dual-process theory was supported.

## Supporting Information

S1 Dataset (XLSX)

S1 Table The Means, Standard Deviations, and Alphas for the Negatively Oriented Items on the Center for Epidemiologic Studies’ Depression Scale (CES-D). (DOCX)

S2 Table Risk Factors of Depressive Symptoms, Assessed Using the 16-item Version of the Center for Epidemiologic Studies’ Depression Scale (CES-D), with a Cut-off of 13. (DOCX)
