# Supplementary material for: The Impact of Coping Flexibility on the Risk of Depressive Symptoms
Source: PLoS One. 2015 May 26;10(5):e0128307. doi: 10.1371/journal.pone.0128307 (PMC4444128; doi:10.1371/journal.pone.0128307)
Supplement: S1 Table — (DOCX) [file pone.0128307.s002.docx]

Supporting Information 1

The Means, Standard Deviations, and Alphas for the Negatively Oriented Items on the Center for Epidemiologic Studies’ Depression Scale (CES-D)

|  |  |  |  |  |  |  |  |  |  |  |
| --- | --- | --- | --- | --- | --- | --- | --- | --- | --- | --- |
|  |  |  |  | 95% CI | |  |  |  | 95% CI | |
|  |  |  |  |  |  |  |  |  |  |  |
| Gender | Mean | SD | Range | LL | UL | Alpha | Range | Prevalence | LL | UL |
|  |  |  |  |  |  |  |  |  |  |  |
| Men | 13.32 | 10.13 | 0-48 | 12.56 | 14.08 | 0.90 | 0-48 | 43.92 | 40.20 | 47.51 |
| Women | 14.86 | 10.40 | 0-48 | 14.24 | 15.48 | 0.91 | 0-48 | 51.15 | 48.17 | 54.37 |
|  |  |  |  |  |  |  |  |  |  |  |

*Note*. CI is confidence interval for OR; LL is lower limit; UL is upper limit. Prevalence is probability using a 13 score as the cut-off point.
