# Supplementary material for: The Impact of Coping Flexibility on the Risk of Depressive Symptoms
Source: PLoS One. 2015 May 26;10(5):e0128307. doi: 10.1371/journal.pone.0128307 (PMC4444128; doi:10.1371/journal.pone.0128307)
Supplement: S2 Table — (DOCX) [file pone.0128307.s003.docx]

Supporting Information 2

Risk Factors of Depressive Symptoms, Assessed Using the 16-item Version of the Center for Epidemiologic Studies’ Depression Scale (CES-D), with a Cut-off of 13

|  |  |  |  |  |  |  |  |  |
| --- | --- | --- | --- | --- | --- | --- | --- | --- |
|  |  |  |  |  |  |  | 95% CI | |
|  |  |  |  |  |  |  |  |  |
| Risk Factor | | B | SE | Wald | OR | *p* value | LL | UL |
|  |  |  |  |  |  |  |  |  |
| Gender | |  |  |  |  |  |  |  |
|  | Men |  |  |  | 1.00 |  |  |  |
|  | Women | 0.26 | 0.10 | 6.42 | 1.29 | 0.011 | 1.06 | 1.58 |
| Coping flexibility | |  |  |  |  |  |  |  |
|  | Evaluation coping | - 0.15 | 0.02 | 77.79 | 0.86 | < 0.001 | 0.83 | 0.89 |
|  | Adaptive coping | - 0.06 | 0.02 | 15.09 | 0.94 | < 0.001 | 0.92 | 0.97 |
|  |  |  |  |  |  |  |  |  |

*Note*. OR is odds ratio; CI is confidence interval for OR; LL is lower limit; UL is upper limit.
